# Promiscuous, Multi-Target Lupane-Type Triterpenoids Inhibits Wild Type and Drug Resistant HIV-1 Replication Through the Interference With Several Targets

**DOI:** 10.3389/fphar.2018.00358

**Published:** 2018-04-18

**Authors:** Luis M. Bedoya, Manuela Beltrán, Javier García-Pérez, Patricia Obregón-Calderón, Oliver Callies, Ignacio A. Jímenez, Isabel L. Bazzocchi, José Alcamí

**Affiliations:** ^1^Retrovirus Laboratory, Department of AIDS Immunopathogenesis, National Centre of Microbiology, Instituto de Salud Carlos III, Madrid, Spain; ^2^Department of Pharmacology, Pharmacy Faculty, Universidad Complutense de Madrid, Madrid, Spain; ^3^Departamento de Química Orgánica, Instituto Universitario de Bio-Orgánica Antonio González, Universidad de La Laguna, San Cristóbal de La Laguna, Spain

**Keywords:** HIV-1, antiretrovirals, maturation, triterpenoids, lupanes, promiscuous compounds, multi-target compounds

## Abstract

Current research on antiretroviral therapy is mainly focused in the development of new formulations or combinations of drugs belonging to already known targets. However, HIV-1 infection is not cured by current therapy and thus, new approaches are needed. Bevirimat was developed by chemical modification of betulinic acid, a lupane-type pentacyclic triterpenoid (LPT), as a first-in-class HIV-1 maturation inhibitor. However, in clinical trials, bevirimat showed less activity than expected because of the presence of a natural mutation in Gag protein that conferred resistance to a high proportion of HIV-1 strains. In this work, three HIV-1 inhibitors selected from a set of previously screened LPTs were investigated for their targets in the HIV-1 replication cycle, including their maturation inhibitor effect. LPTs were found to inhibit HIV-1 infection acting as promiscuous compounds with several targets in the HIV-1 replication cycle. LPT12 inhibited HIV-1 infection mainly through reverse transcription, integration, viral transcription, viral proteins (Gag) production and maturation inhibition. LPT38 did it through integration, viral transcription or Gag production inhibition and finally, LPT42 inhibited reverse transcription, viral transcription or Gag production. The three LPTs inhibited HIV-1 infection of human primary lymphocytes and infections with protease inhibitors and bevirimat resistant HIV-1 variants with similar values of IC_50_. Therefore, we show that the LPTs tested inhibited HIV-1 infection through acting on different targets depending on their chemical structure and the activities of the different LPTs vary with slight structural alterations. For example, of the three LPTs under study, we found that only LPT12 inhibited infectivity of newly-formed viral particles, suggesting a direct action on the maturation process. Thus, the multi-target behavior gives a potential advantage to these compounds since HIV-1 resistance can be overcome by modulating more than one target.

## Introduction

Drug discovery has been classically focused on the development of highly selective single-target drugs in order to minimize undesired side effects. However, the ever-increasing rate of failure during late-stage clinical development is changing the drug discovery landscape by considering new strategies (Senger et al., 2016). Compound promiscuity (Hu and Bajorath, 2013) which provides polypharmacology drug behavior is a main concern in drug discovery (Morphy and Rankovic, 2005) and a number of computational approaches (Sturm et al., 2012; Abdolmalek et al., 2017) and NMR (LaPlante et al., 2013) strategies have been introduced to understand the molecular basis for drug promiscuity. Furthermore, multi-target drugs have become a hotspot in new drug research and development, showing themselves to be effective in reducing the likelihood of drug resistance, diminishing problems of dosing complexity, drug-drug interactions and toxicities, as well as improving patient compliance. Multi-target drugs offer an advantage in the treatment of certain complex diseases by modulating more than one target, and this approach becomes especially relevant in complex (multi-factorial) diseases such as cancer and AIDS (Zhan and Liu, 2009).

Lupane-type pentacyclic triterpenoids (LPTs) have been extensively studied as anti-HIV agents, providing a versatile structural platform for the discovery of new biologically active compounds, and thus, the molecular structure of the LPTs could be considered as a *privileged structure* since they can be recognized by distinct receptors, leading to a broad range of pharmacological activities (Vasilevsky et al., 2011). In particular, LPTs have been extensively studied as anti-HIV agents, providing a versatile structural platform for drug discovery. Furthermore, the LPTs named lupeol, betulin, and betulinic acid are reported to possess several biological properties, including anticancer, anti-inflammatory and antiviral (HIV-1) (Cichewicz and Kouzi, 2004). One of the most well-known member of the lupane family is betulinic acid [BA, 3β-hydroxy-lup-20(29)-en-28-oic acid], a LPT found in abundance in many plant species.

In the last 15 years, many BA derivatives bearing a side-chain modification at C-28 or/and C-3 position have been reported to inhibit HIV-1 replication (Qian et al., 2009). In fact, bevirimat (BVM) [3-*O*-(3′, 3′-dimethylsuccinyl)betulinic acid] is a first-in-class HIV-1 maturation inhibitor (MI) (Li et al., 2003). However, BVM failed in phase IIb clinical trials due to naturally occurring polymorphisms in HIV-1 Gag leading to a natural resistance of the virus to the drugs (Lu et al., 2011). A number of BVM analogs with increased hydrosolubility (Coric et al., 2013) or with an improved antiviral profile (Zhao et al., 2016), in addition to insights into the structural and functional requirements for HIV-1 maturation inhibition (Lin et al., 2016; Tang et al., 2017) have been reported. Currently, BMS-955176, a second generation MI with Gag polymorphic coverage, is in phase IIb clinical trials (Nowicka-Sans et al., 2016).

**Figure 1:**
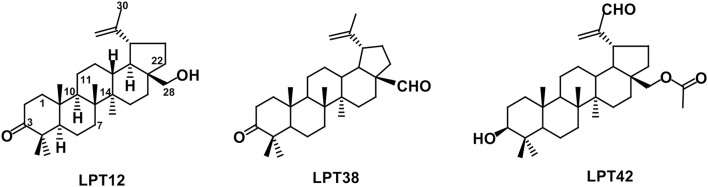
Chemical structures of LPT12, LPT38 and LPT42.

Despite emerging drug resistance against antiretroviral therapy, antiretroviral drugs which are currently in development still target the same steps of the HIV-1 replication cycle than those antiretroviral drugs which are currently used in the clinic (Gravatt et al., 2017). Therefore, new drugs targeting different steps in the viral replication cycle may help to overcome drug resistance. In previous research from our group, a series of terpenes of the oleanane and lupane families have demonstrated potent anti-HIV-1 activity (Osorio et al., 2012; Callies et al., 2015). Among the lupane triterpenoids, the 28-hydroxy-3-oxo-lup-20(29)-ene (betulone), 3-oxo-lup-20(29)-en-28-al and 28-acetoxy-3β-hydroxy-lup-20(29)-en-30-al were selected for future studies (Figure 1). In this work, the mechanism of action of these three potent anti-HIV-1 lupane triterpenoids has been investigated for their interaction in different steps of the HIV-1 replication cycle.

Although the structural differences among these compounds are very subtle, these slight structural variations have the potential to modulate the affinity for certain targets in the viral replication cycle or even to target several steps or different targets of the HIV-1 replication cycle, leading to novel mechanisms of action.

## Materials and methods

### Chemistry

The natural compound LPT12 (betulone) was isolated from the root bark of *Maytenus cuzcoina* (*Celastraceae*). Analogues LPT38 [3-oxo-lup-20(29)-en-28-al] and LPT42 [28-acetoxy-3β-hydroxy-lup-20(29)-en-30-al] were prepared from betulin and 28-*O*-acetylbetulin, respectively, as previously described (Callies et al., 2015).

### Cells

The human lymphocyte cell line, MT-2 (Harada et al., 1985) was cultured in complete RPMI 1640 medium containing 10% (v/v) fetal bovine serum, 2 mM L-glutamine, penicillin (50 IU/mL) and streptomycin (50 μg/mL) (all Whittaker M.A. Bio-Products, Walkerville, MD). The human kidney derived cell line, 293T, was obtained from the American type culture collection ATCC ref: ATCC® CRL-3216™ and the cervix derived Hela-Tet-On-Luc, which contains a pTET-ON construct which, in response to doxycycline, gets active and starts the synthesis of luciferase gene, was kindly provided by Dr. Eduardo Muñoz of Cordoba University. Both cell lines were cultured in complete DMEM medium containing 10% (v/v) fetal bovine serum, 2 mM L-glutamine, penicillin (50 IU/mL), and streptomycin (50 μg/mL) (all Whittaker M.A. Bio-Products, Walkerville, MD). HeLa-Tet-On-Luc cell line was maintained in complete DMEM medium in the presence of 100 μg/mL of hygromycin and 100 μg/mL of G418 (Both Life Technologies). PBMCs (peripheral blood mononuclear cells) were isolated from healthy blood donors by centrifugation through a Ficoll-Hypaque gradient (Pharmacia Corporation, North Peapack, NJ) and were resuspended in RPMI 1640 medium supplemented with 10% fetal bovine serum, 2 mM L-gutamine and antibiotics (100 mg/L streptomycin and 100 U/mL penicillin) (all Whittaker M.A. Bio-Products, Walkerville, MD, USA) before culture at a concentration of 2 × 10^6^ cells/mL. PBLs were obtained using human CD4 MicroBeads (Biotec) and activated with Il-2 (300 IU/mL Chiron) for at least 48 h. Blood samples (Buffy coat) were obtained from the Transfusion Center of the Community of Madrid (Spain). Proper informed consent was obtained from each subject in accordance with the Spanish legislation on blood donor regulations. Confidentiality and privacy was assured. All cells were cultured at 37°C in a 5% CO_2_ humidified atmosphere and subcultured twice per week.

### Viruses and constructs

The vector pNL4.3-Luc was generated by cloning the *luciferase* gene in the HIV-1 proviral clone pNL4.3. Construct pNL4.3-Ren was generated by cloning the *renilla* gene in the Luc site of pNL4.3-Luc (Garcia-Perez et al., 2007) and pJR-Ren construct was generated by cloning the *env* gene of HIV-1 JR_CSF_ in the pNL4.3-Ren construct (González et al., 2010). NL4.3-Δ-env-VSV-Luc (VSV-Luc) supernatants were obtained by co-transfection of pNL4.3-Luc-R^−^E^−^, a full length HIV-1 DNA that do not express HIV-1 envelope obtained from NIH (AIDS Research and Reference Reagent Program, NIAID, NIH), and pcDNA-VSV, DNA for vesicular stomatitis virus (VSV) G glycoprotein cloned in the pcDNA3.1 construct (Oberlin et al., 1996). V370A mutation was introduced by PCR in pNL4.3-Ren clone using the QuickChange Lightning Site-Directed Mutagenesis Kit (Agilent Technologies, Santa Clara, CA, USA) to produce replication-competent virus (NL-V370A-Ren, mutation V370A) expressing the *renilla* reporter gene. pNL4.3-2169-Ren is extensively described by Garcia-Perez et al. (2007) (PI mutations: M36I, I54V, L63P, A71V, G73S, L90M).

### Recombinant virus assay (RVA)

Evaluation of the anti-HIV-1 activity of compounds was performed in a biosecurity safety level (BSL) 2/3 laboratory in the Centro Nacional de Microbiología, ISCIII as follows: infectious supernatants were obtained from 293T cells transfected with the pNL4.3-Ren construct alone or co-transfection of pNL4.3-Luc-R- E- and pcDNA3-VSV constructs. All transfections were carried out with calcium phosphate. Briefly, 293T cells (5·10^6^) were seeded in culture flask (T75 cm^2^) 24 h before the transfection. DNA (40 μg) were then mixed with Cl_2_Ca (0.75 mL) and HBS buffer (50 mM HEPES, 1.5 mM Na_2_HPO_4_, 140 mM NaCl, pH 7.05) (0.75 mL) and added to the culture. After 18 h, culture media is changed for fresh DMEM and 24 h later supernatants were collected. These supernatants were used to infect cells in the presence or absence of the compounds to evaluate anti-HIV-1 activity. Luminescence was quantified 48 h post-infection. Briefly, cells were lysed with 100 μl of buffer provided by “Luciferase Assay System Kit with Reporter Lysis Buffer” or “Renilla Assay System” (both Promega, Madison, WI, USA). Relative luminescence units (RLUs) were obtained in a luminometer (Berthold Detection Systems, Pforzheim, Germany) after the addition of substrate to cell extracts. Viability was performed in parallel treated cells with the same concentrations of compound as in the RVA. After 48 h, cell viability was evaluated with the CellTiter Glo assay system (Promega, Madison, WI, USA) following the manufacturer's specifications. Inhibitory concentrations 50 (IC_50_) and cytotoxic concentrations 50% (CC_50_) were calculated using GraphPad Prism Software.

### Specificity assay

The Hela-Tet-On-Luc cell line contains two constructs: the pTET-ON codifies constitutively for rtTA protein that, in response to doxycycline, gets active and binds to the pTRE2hyg-Luc starting the synthesis of luciferase gene mRNA leading to luciferase protein expression. Briefly, cells (10^5^ cells/mL) were seeded the day before the assay, and then stimulated with doxycycline (BD Bioscience Clonetech) at 1 μg/mL in the presence or absence of LPTs for 6 h. Then, cells were washed twice in PBS, lysed and the luciferase activity measured in a luminometer.

### Quantification of early and late reverse transcription

MT-2 cells were pre-treated with different concentrations of LPTs and infected with a NL4.3 wild type HIV-1 for 5 h. Compound concentrations were chosen on the basis of their previously observed EC_50_ and CC_50_ values. Total genomic DNA was isolated with a QIAamp DNA blood mini kit, Qiagen (Qiagen, Hilden, Germany) and quantified with a Nanodrop-1000 spectrophotometer (ThermoFisher, Wilmington USA).

Early and late viral DNAs were quantified by qPCR as previously described (Bermejo et al., 2015). Briefly, 50–100 ng DNA were mixed with 1 μM forward and reverse primers (MA pr-243 and MA pr-244, and MA pr-275 probe for early RT and primers MH531 and MH532 and LRT-P probe for late RT) (Table S1) and 1x GoTaq Probe Universal Master Mix (Promega, Madison, WI, USA) in a final volume of 16 μL. qPCR was performed in triplicate in a StepOne Real-Time PCR system (Life Technologies) using standard cycling conditions. Serial dilutions of genomic DNA from 8E5 cell line, which contain a single integrated copy of HIV-1 (Folks et al., 1986), were used as standard curve. The CCR5 gene was used as endogenous control (primers CCR5_F and CCR5_R and CCR5 probe in Table S1). Cell viability was evaluated in mock infected cells treated with LPTs following the same conditions of the assay.

### Quantification of proviral integration

Integrated proviral DNA was quantified within whole genomic DNA from 5 days post-infection. Whole genomic DNA from HIV-infected MT-2 cells treated with LPTs or controls was analyzed by nested Alu-LTR PCR, as previously described (Brussel and Sonigo, 2003; Table S1) using a StepOne Real-Time PCR System (Life Technologies). In brief, a first conventional PCR was performed using oligonucleotides against Alu sequence and the HIV-1 LTR forward primers (Alu 1 and Alu 2, and reverse primer L-M667 in the Table S1). Then, a second qPCR was performed using primers Lambda T and AA55M and Taqman probe MH603 (Table S1) with FAM/ZEN/Iowa Black (Integrated DNA Technologies) and 1x GoTaq Probe Universal Master Mix (Promega, Madison, WI, USA). Serial dilutions of genomic DNA from 8E5 cell line, which contain a single integrated copy of HIV-1, were used as standard curve. The CCR5 gene was used as endogenous control (primers CCR5_F and CCR5_R and CCR5 probe in Table S1). Cell viability was evaluated in mock infected cells treated with LPTs following the same conditions of the assay.

### Transfection assays

MT-2 cells were maintained in culture without stimuli for 1 day prior to the assay. Cells were then suspended in 350 μl of RPMI without serum and antibiotics and added to a 4 mm cuvette with 1 μg/10^6^ cells of the construct pNL4.3-Luc. Afterwards, the mixture was transfected/pulsed at 280 V, 1.500 μF and maximum resistance using an Easyject plus Electroporator (Equibio, Middlesex, UK). After transfection, cells were immediately cultured in RPMI with 10% fetal bovine serum and antibiotics and treated with LPTs at active concentrations. Cell cultures were harvested 48 h later and luciferase activity (RLUs) measured in a luminometer (Berthold Detection Systems, Pforzheim, Germany).

### Maturation assay

293T cells were seeded in 96 microwell plates and the DMEM was replaced with fresh media 4 h before the experiment. The pNL4.3-Luc construct was then transfected in each well using the calcium phosphate method as described above. The DMEM was replaced 18 h later and either LPTs or vehicle (DMSO) were added at different concentrations; half of the wells were left untreated. The supernatants were collected 48 h after transfection and the p24 Gag protein was quantified. Then, the supernatants were used to infect MT-2 cells, controlling for p24 protein amount (10 ng/well) to avoid any pre-maturation effects on viral particle production. This assay was performed with selected concentrations of LPTs and bevirimat was used as a control. LPTs were still present in viral supernatants used to infect MT-2, but diluted to the final infectious titre, which was around 10 times in our experiments. The active fraction of LPT present in the supernatant was therefore also diluted below IC_10_ levels. To assure that replication inhibition was not due to a residual activity, a control of dilution was used with MT-2 cells infected with untreated supernatants in the presence of final concentrations of 1/10 diluted LPTs.

### Western blot and preparation of extracts

293T cells were transfected with approximately 20 ng of HIV-1 NL4.3 construct and incubated in the presence of LPTs for 24 h at 37°C. Supernatants were collected and submitted to several rounds of concentration by ultra-centrifugation. Total proteins were extracted from these concentrated supernatants in 25 μl of lysis buffer (20 mM Hepes pH 8.0, 10 mM KCl, 0.15 mM EGTA, 0.15 mM EDTA, 0.5 mM Na_3_VO_4_, 5 mM NaFl, 1 mM DTT, leupeptin 1 mg/mL, pepstatin 0.5 mg/mL, aprotinin 0.5 mg/mL, and 1 mM PMSF) containing 0.5% NP-40. Protein concentration was determined by the Bradford assay (Bio-Rad, Richmond, CA, USA) and 30 μg of proteins were boiled in loading buffer and electrophoresed in 12.5% SDS/polyacrylamide gels at 100 V for 2 h. Separated proteins were transferred to nitrocellulose membranes (32 mA; 4°C) for 2 h. Blots were blocked in TBS solution containing 0.1% Tween-20 and 5% non-fat dry milk overnight at 4°C. Immunodetection of specific proteins was carried out with a monoclonal antibody against HIV-1 Gag protein (HIV-1 p24 Antibody 1941, Dallas, TX, USA). Secondary antibodies conjugated with horseradish peroxidase (HRP) were purchased from GE Healthcare. Proteins were detected with SuperSignal West Pico/Femto Chemiluminescent Substrate (Thermofisher, Rockford IL, USA).

### Statistical analysis

All the inhibitory concentrations 50 (IC_50_) and cytotoxic concentrations 50% (CC_50_) were calculated using non-linear regression, dose-response inhibition curves. ANOVA analysis was used to determine the value of p (^*^p < 0.05, ^**^p < 0.01 ^***^p < 0.001). Both analyses were performed using GraphPad Prism Software.

## Results

### Anti-HIV-1 activity and viral entry inhibition

**Figure 2:**
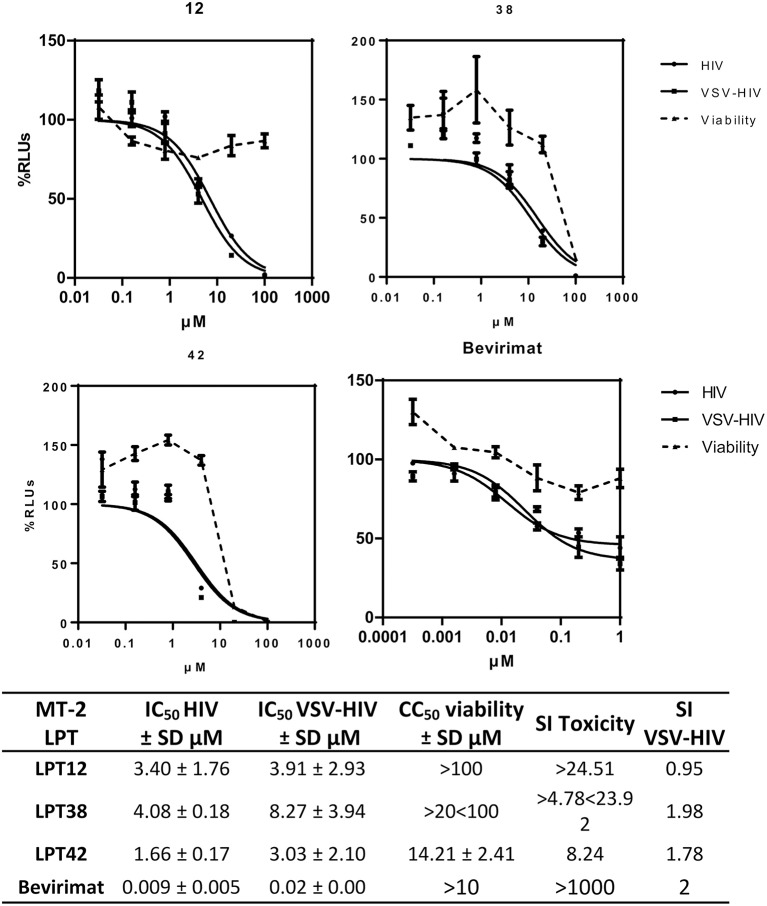
Dose response inhibition curves of HIV-1 (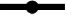
HIV) and VSV-pseudotyped HIV-1 (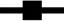
VSV-HIV) infections of MT-2 cells in the presence of LPT12, LPT38, LPT42 (0.1–100 μM), and bevirimat (0.0001–1 μM). Viability was measured in parallel mock infected cell cultures (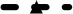
Viability). Results are shown as percentage of RLUs as compared to an untreated control. IC_50_ values were calculated with GraphPad Prism software, using non-linear regression analysis (Log inhibitor vs. response). SD, Standard deviation; SI toxicity, Specificity index toxicity (CC_50_/IC_50_); SI VSV-HIV, (Specificity index IC_50_ VSV/IC_50_ HIV-1).

To evaluate the activity of HIV-1 replication and viral entry, infections with wild type HIV-1 and VSV-pseudotyped HIV-1 were performed in parallel in the presence of different concentrations of LPTs or bevirimat (Figure 2). VSV-pseudotyped HIV-1 is an *env* gene deleted HIV-1 carrying the G protein of VSV instead, and thus, it enters target cells by a CD4/co-receptor independent pathway. Results are the mean ± SD of at least 3 independent experiments, each in triplicate.

The IC_50_s of the three LPTs (Figure 2) were in the micromolar range, between 1 and 5 μM, while bevirimat IC_50_ was in the nanomolar range, as described previously (9 nM). However, LPTs12 and LPT38 were able to inhibit HIV-1 infection with no or slight cell toxicity at the concentrations tested (CC_50_ greater than 100 μM for compound LPT12 and greater than 20 μM for compound LPT38), while compound LPT42 displayed cell toxicity with a CC_50_ of 14.21 μM and an SI of around 8.

Regarding HIV-1 entry, the three LPTs and bevirimat inhibited both infections, wild type HIV-1 and VSV-pseudotyped HIV-1, with similar IC_50_ values, since specificity index (SI VSV-HIV) was close to 1 and graphic representations showed overlapping curves, suggesting an antiviral effect independent of the HIV-1-envelope. LPT12 displayed the best specificity index toxicity/activity (higher than 24) while LPT38 and, in particular, LPT42 showed lower SIs.

### LPTs effects on reverse transcription and integration

**Figure 3:**
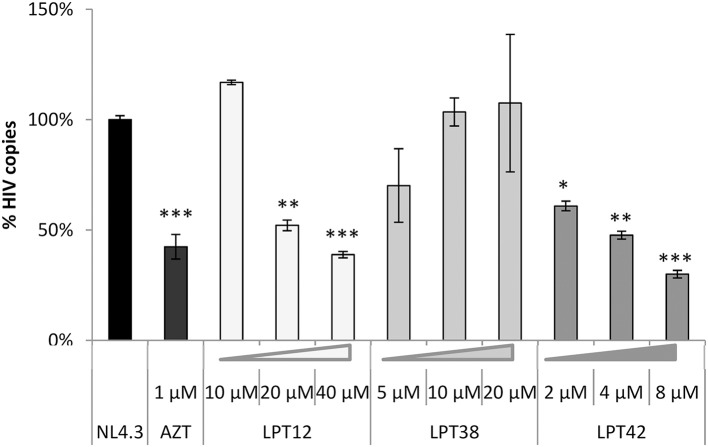
LPTs activity on HIV-1 reverse transcription. Analysis by qPCR of late reverse transcripts in MT-2 cells treated with LPT12 (10, 20, and 40 μM), LPT38 (5, 10, and 20 μM), and LPT42 (2, 4, and 8 μM) and infected with NL4.3 wild type HIV-1 (HIV-1). Results are shown as % of HIV-1 cell copies using 8E5 cells as a reference of the number of HIV-1 copies in cell culture. The untreated control represents the maximum of HIV-1 copies detected (100%). ANOVA analysis was performed to determine the value of *p* (*p < 0.05, **p < 0.01,***p < 0.001) and NL4.3 (untreated HIV-1 infection) was used as control.

**Figure 4:**
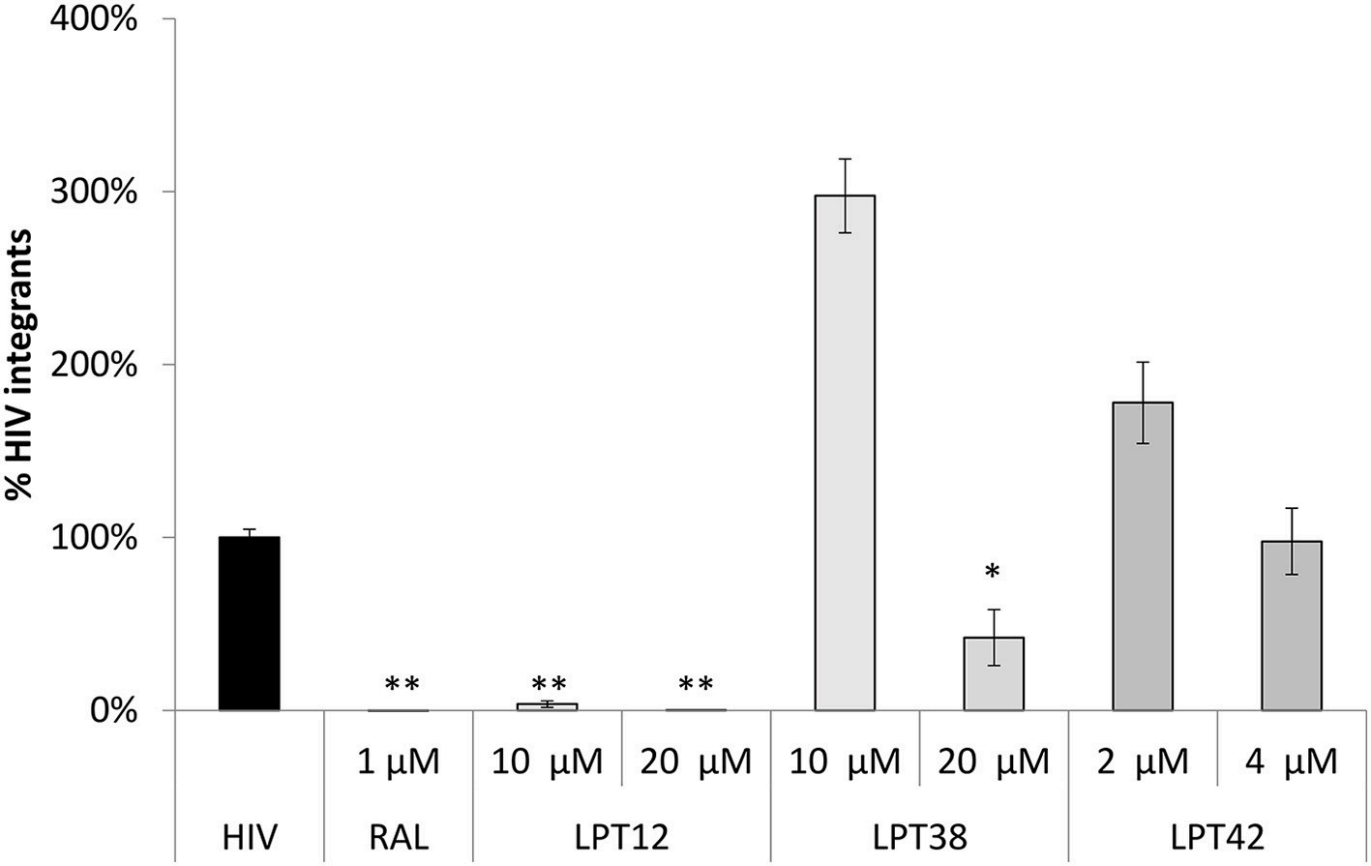
LPTs activity on HIV-1 integration. Analysis by qPCR of HIV integration in MT-2 cells treated with AZT (1 μM), LPT12 (10 and 20 μM), LPT38 (5 and 10 μM) or LPT42 (2 and 4 μM) and infected with NL4.3 wild type HIV-1 (HIV). Results are shown as % of HIV-1 integrants. The untreated control (HIV) represents the maximum of HIV-1 integrants detected (100%). CCR5 was used as housekeeping gene. ANOVA analysis was performed to determine the value of *p* (*p < 0.05, **p < 0.01,***p < 0.001).

After cell entry, HIV reverse transcriptase (RT) transcribes viral RNA to viral DNA, which is transported to the cell nucleus to integrate in the host cell genome by the viral integrase. We have measured both the generation of reverse transcripts and integrated viral DNA in infected cells after the treatment with LPTs at different concentrations. To do that, we have used a quantitative PCR assay to quantify HIV-1 LTR sequences (Figures 3, 4).

The amount of late DNA viral transcripts was decreased by AZT (zidovudine) as compared to a non-treated control (HIV-1, 100%). LPT12 and LPT42 also diminished the amount of viral transcripts at the concentrations tested while LPT38 did not.

Raltegravir (RAL) was able to block almost completely proviral DNA integration (Figure 4). We found a different pattern of integrase inhibition among LPTs, with LPT12 as the most powerful integrase inhibitor (complete inhibition at 10 μM). LPT38 decreased the amount of integrated copies of HIV-1 only at 20 μM. LPT42 did not show any effect on integrase activity at the concentrations tested (2 and 4 μM) although a slight tendency could be noted. We increased LPT42 concentration to explore this tendency, but this experiment is performed at 5 days, treating every 2 days with LPTs, and LPT42 displayed cell toxicity at the higher concentration used of 8 μM (its CC_50_ was 14.2 μM at 48 h). Interestingly, LPT38 and LPT42 at low concentrations (10 and 2 μM) were able to enhance HIV-1 integration.

Therefore, we found that LPT12 was able to inhibit both steps, reverse transcription and integration, decreasing the amount of reverse transcripts at 20 and 40 μM and total integrated viral DNA at both concentrations tested (10 and 20 μM). LPT38 results showed more variability than any of the other samples, although it was clear that it decreased the amount of integrated viral DNA (20 μM) and it did not decrease the amount of DNA reverse transcripts at the concentrations tested. On the other hand, LPT42 decreased the amount of viral DNA reverse transcripts but did not decrease viral integrated DNA. To rule out low DNA detection due to LPTs toxicity, cell viability was evaluated in mock infected cells treated with LPTs following the same conditions of the assay and non-toxic concentrations were used (data not shown). All the experiments are the mean +/- SD of at least 3 independent experiments, each in duplicates.

### LPTs effects on post-integration targets: viral transcription, protein expression and maturation

**Figure 5:**
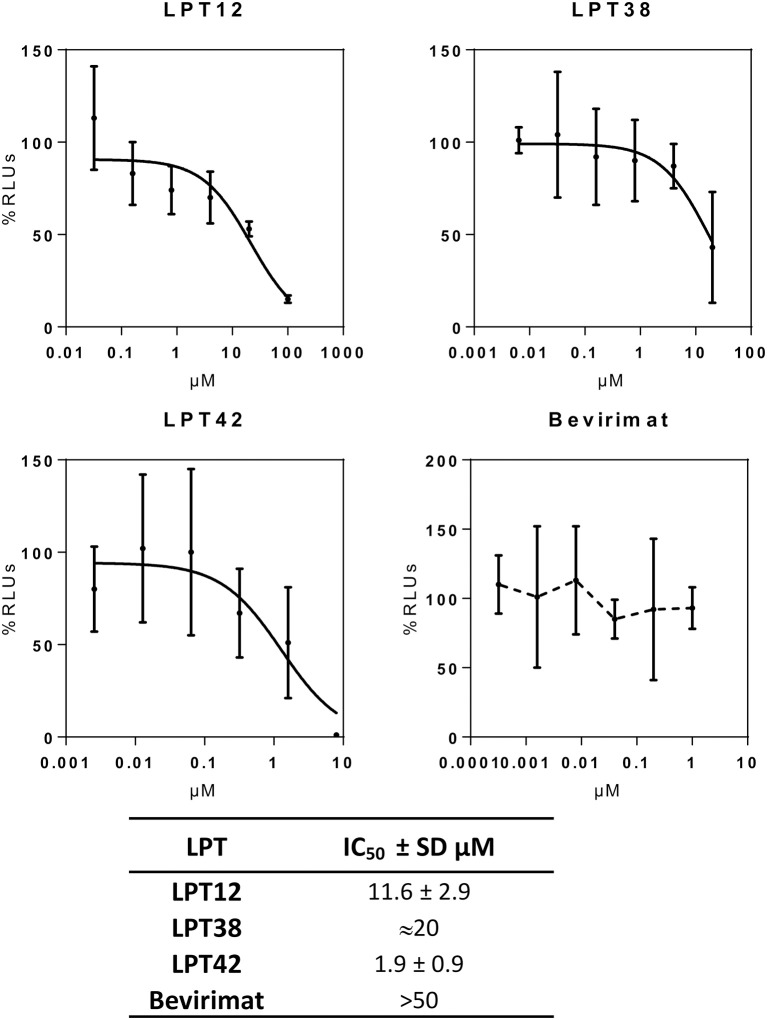
HIV-1 transcriptional activity of selected LPTs. MT-2 cells were transfected with a luciferase construct under the control of the whole HIV-1 genome (pNL4.3-Luc) in the presence of different concentrations of LPTs or bevirimat. Data are shown as % RLUs as compared to an untreated control (100%). Concentrations are shown in μM. IC_50_s were calculated using GraphPad Prism software. SD, Standard deviation.

To evaluate the effect on viral transcription, the whole genome of HIV-1 (NL4.3-Luc) was transfected in MT-2 cells in the presence or absence of effective concentrations of LPTs. Thus, all the steps previous to viral transcription are bypassed and it is possible to evaluate separately the post-integration effect of LPTs. Bevirimat was used as treatment control (Figure 5).

**Figure 6:**
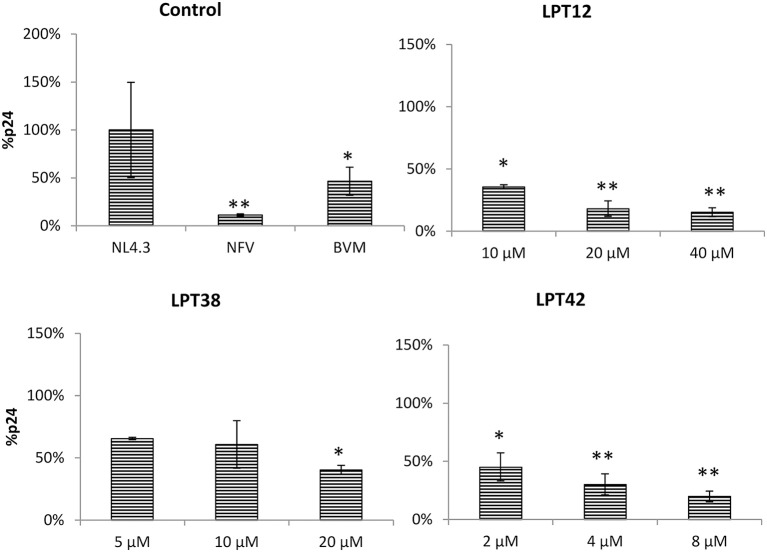
Inhibition of HIV-1 (NL4.3) Gag production in 293T cells by LPT12, LPT38, LPT42, Nelfinavir 0.1 μM (NVF), and Bevirimat 0.1 μM (BVM). p24 Gag protein were detected by an ELISA Elecsys system (Roche). Results are shown as percentage of Gag-p24 protein as compared to an untreated control (100%). ANOVA analysis was performed to determine the value of *p* (*p < 0.05, **p < 0.01) and NL4.3 (untreated HIV-1 transfection) was used as control.

LPT12 inhibited viral transcription with an IC_50_ of 11.6 μM, which is almost 3 fold higher than its IC_50_ for HIV-1 infection inhibition (3.4 μM). LPT42 instead showed an IC_50_ of 0.4 μM, 10 fold lower than its IC_50_ for HIV-1 infection, and thus, HIV-1 transcription could be one of its main targets in the viral replication cycle. LPT38 was able to inhibit HIV-1 transcription with an IC_50_ of approximately 20 μM, 5 fold higher than its IC_50_ in infection (4.18 μM). Moreover, LPT38 CC_50_ is greater than 20 and lower than 100 μM, and thus, transcriptional inhibition could be a consequence of cell toxicity, at least in part. As expected, bevirimat does not alter HIV-1 transcription. After transcription, viral mRNAs are transported to the cell cytoplasm and are translated into proteins that will be processed by the viral protease. Several antiretroviral drugs target the HIV-1 protease (protease inhibitors or PIs) and this could also be a potential target for LPTs. Thus, the whole genome of HIV-1 (NL4.3-Luc) was transfected in 293T cells in the presence or absence of effective concentrations of LPTs, supernatants were collected and Gag p24 protein measured by ELISA (Figure 6).

LPTs diminished p24 protein detection as compared to a non-treated control at all the concentrations tested as well as the protease inhibitor nelfinavir. Interestingly, bevirimat was also able to lower p24 production, suggesting that inhibition of maturation decreased p24 production. However, this effect could be due to the inhibition of HIV-1 transcription and not to a direct effect on Gag synthesis.

To study the effect of LPTs on the morphogenesis of new viral particles, 293T cells were transfected with a construct encoding the whole genome of wild type HIV-1 (NL4.3-Luc) or bevirimat resistant HIV-1 (NL4.3-V370A-Luc) and treated with LPTs, nelfinavir or bevirimat. Supernatants from transfected cells were collected and the amount of p24 Gag protein measured. Afterwards, these supernatants were used to infect MT-2 cells with the same amount of Gag protein. Thus, any effect of LPTs on morphogenesis will produce less infectivity of the treated supernatant as compared to a non-treated control. Since compounds cannot be removed from viral supernatants, infections with dilution controls were performed to rule out a residual activity of compounds.

**Figure 7:**
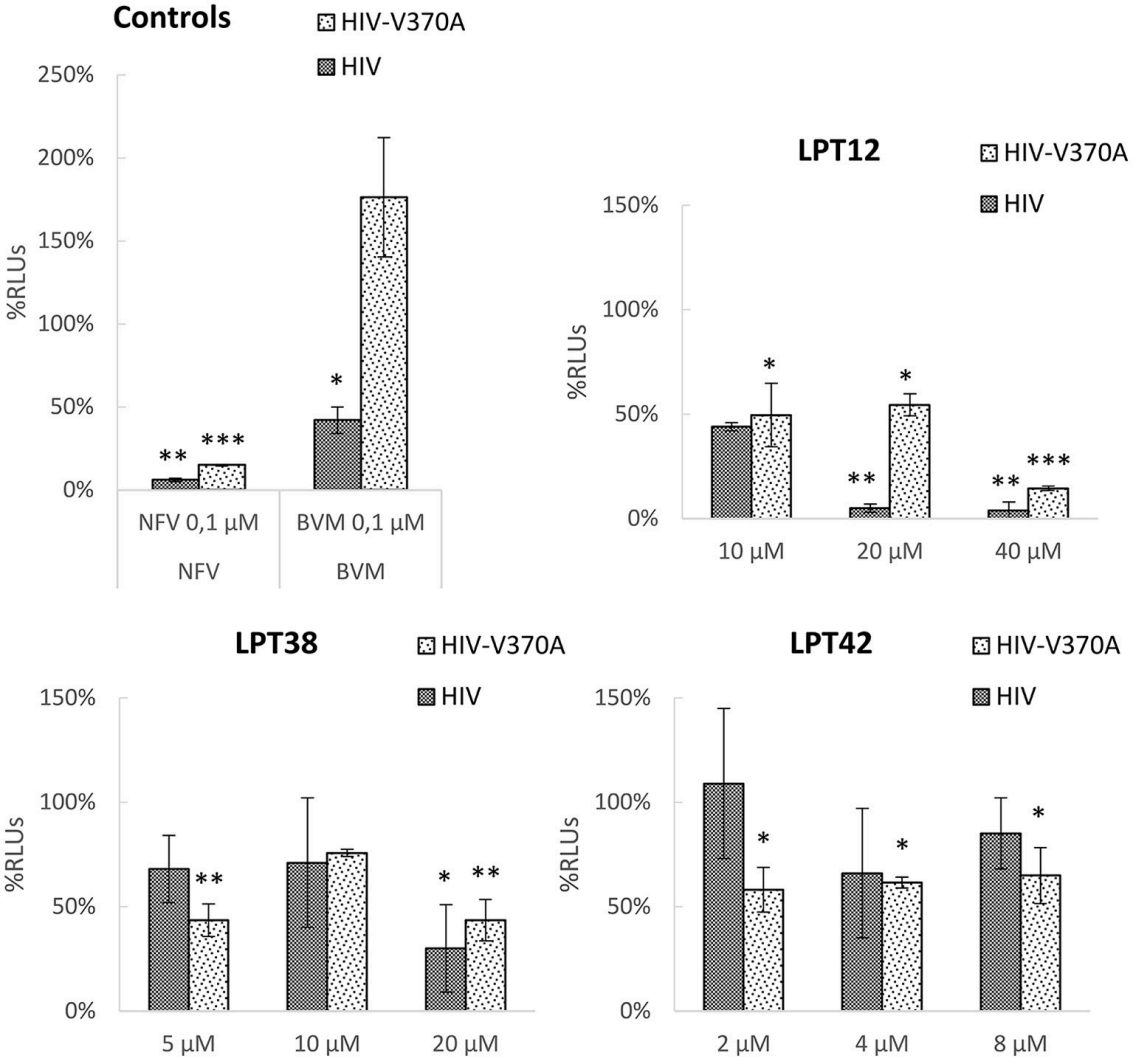
HIV-1 maturation activity of LPTs and bevirimat. 293T cells were transfected with pNL4.3-Luc or pNL4.3-V370A-luc encoding the whole genome of wild type HIV-1 or bevirimat resistant HIV-1 in the presence of different concentrations of LPT12, LPT38, and LPT42 and controls nelfinavir (NFV) and bevirimat (BVM). Supernatants were then collected, p24 Gag protein quantified and used to infect MT-2 cells with the same amount of p24. Results are shown as percentage of RLUs as compared to a non-treated control (100%). ANOVA analysis was performed to determine the value of *p* (*p < 0.05,**p < 0.01, ***p < 0.001) and NL4.3 (untreated HIV-1 supernatants infection) was used as control.

All the LPTs inhibited HIV-1 infectivity, although differences in their potency are easily appreciated (Figure 7). The effect of both LPT38 and LPT42 on maturation remained around 50% even at the higher concentrations tested. Moreover, LPT12 was able to reduce HIV-1 wild type infectivity to 50% at 10 μM and almost completely at 20 and 40 μM. However, LPT12's effect on bevirimat-resistant HIV-1 (HIV-V370A) seems to be less potent, since the effect at 20 μM remained at 50%, while at 40 μM it was able to inhibit infectivity completely. HIV-1 inhibition by LPTs diluted to the remaining concentrations in the maturation assay was not detected (data not shown). Results are the mean +/- SD of at least 3 independent experiments, each in triplicate.

### Protease-resistant HIV-1 is also susceptible to LPTs inhibition

Protease inhibitors (PIs) are a class of antiretroviral drugs used in ART, although resistances and drug related toxicity have limited their use. Since LPTs are able to inhibit p24-Gag protein production, we have investigated their effect on HIV-1 strains resistant to current PIs.

**Figure 8:**
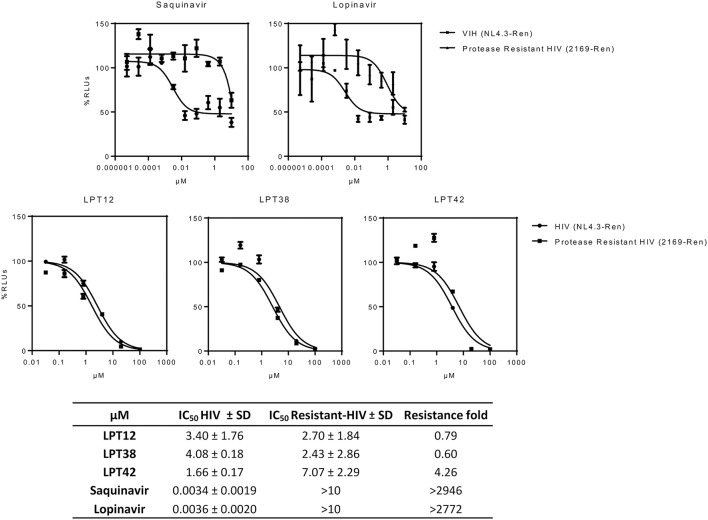
Effect of LPTs on MT-2 infection with protease inhibitors resistant HIV-1 (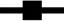
2169-Ren) as compared to the wild type recombinant virus (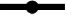
HIV NL4.3-Ren). Results are shown as percentage of RLUs as compared to an untreated control. IC_50_ values were calculated with GraphPad Prism software, using non-linear regression analysis (Log inhibitor vs. response). SD, Standard deviation.

LPTs were able to inhibit saquinavir and lopinavir-resistant HIV-1 with similar or even lower IC_50_s than wild type HIV-1. Only LPT42 showed a slight increase (4.26 fold) in its IC_50_ when a protease inhibitor resistant HIV-1 was used (Figure 8). Results are shown as the mean +/- SD of at least 3 independent experiments, each in triplicate.

### Bevirimat-resistant HIV-1 is susceptible to LPTs inhibition

**Figure 9:**
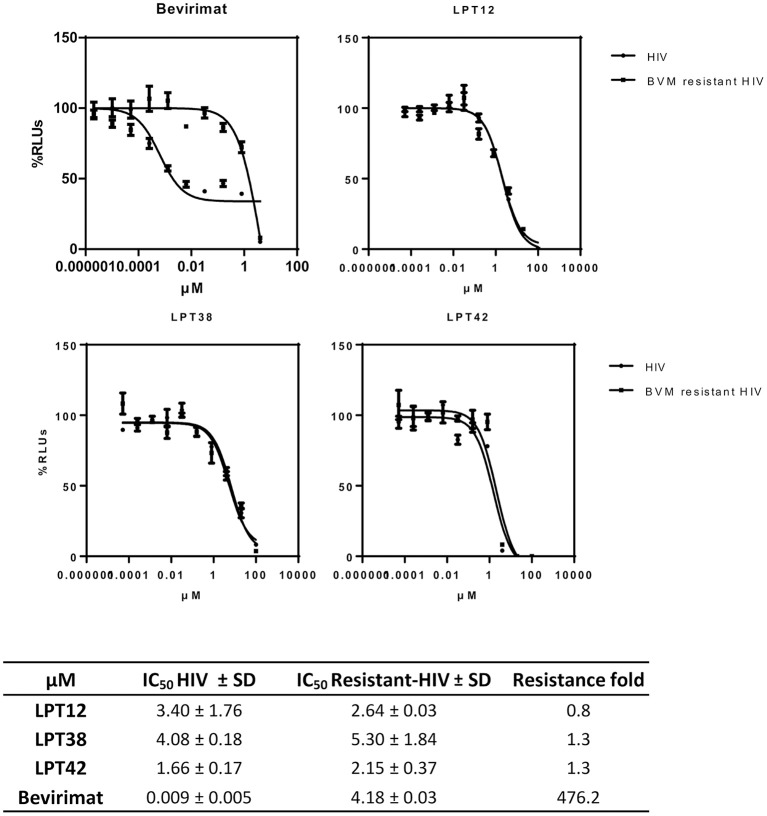
Effect of LPTs on MT-2 infection with a bevirimat-resistant HIV-1 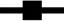
BVM resistant HIV) as compared to the wild type virus (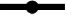
HIV). Results are shown as percentage of RLUs as compared to an untreated control. IC_50_ values were calculated with GraphPad Prism software, using non-linear regression analysis (Log inhibitor vs. response). SD, Standard deviation.

As mentioned above, the natural polymorphism in the *gag* gene occurring in several HIV-1 strains lead to bevirimat-resistance. This was the reason why a further pharmaceutical development of bevirimat as an antiretroviral drug was abandoned. Therefore, in order to evaluate if LPTs can overcome bevirimat resistance, we tested the antiviral activity of LPTs and bevirimat as a control in infections with bevirimat resistant HIV-1 (NL4.3-V370A-Ren), as compared to wild type recombinant virus (NL4.3-Ren) (Figure 9). Results are the mean +/- SD of at least 3 independent experiments, each in triplicate.

The LPTs were able to inhibit both wild type HIV-1 and bevirimat resistant HIV-1 strains with similar values of IC_50_, whereas bevirimat resistance was greater than 450 fold. Thus, LPTs activity is not altered by the presence of the V370A mutation, and thus, LPTs can overcome the resistance to bevirimat due to their multitarget profile.

### Inhibition of processing of HIV-1 gag proteins by LPTs

293T cells were treated with the higher non-toxic concentration of LPTs, bevirimat or nelfinavir and transfected with a wild type HIV-1 (NL4.3). Supernatants were then collected for western blot analysis 2 days after transfection. Accumulation of CA-SP1 (p25) was analyzed using a monoclonal antibody against p24 CA antigen. The signature event of anti-maturation activity is a partial inhibition of CA-SP1 (p25) cleavage that occurs without interfering with other Gag cleavage sites. Inhibition of CA-SP1 cleavage should result in an accumulation of p25.

**Figure 10:**
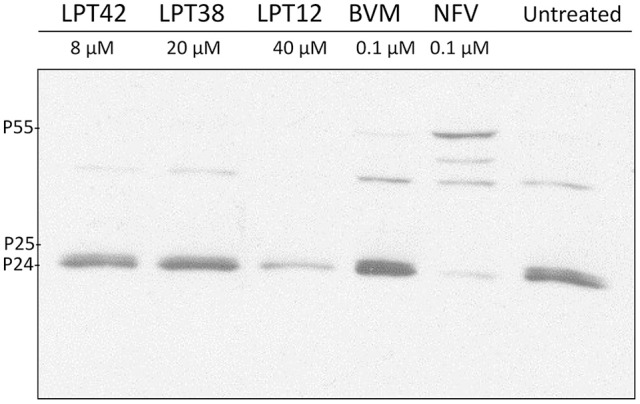
Detection of p24 processing in wild type NL4.3 HIV-1 strain after treatment with LPTs. 293T cells were transfected with HIV-1 (NL4.3) and incubated in presence of LPT12 40 μM, LPT38 20 μM, LPT42 8 μM, bevirimat (BVM) 0.1 μM, nelfinavir (NFV) 0.1 μM or left untreated for 24 h at 37°C. Two days after transfection supernatants were collected and submitted to several rounds of concentration by ultra-centrifugation. Total proteins were then extracted and immunodetection of specific proteins was carried out with a monoclonal antibody against HIV-1 Gag p24 antigen.

We did not detect differences among the untreated control and LPT42 and LPT38 treated cells, suggesting a lack of effect on CA-SP1 processing. As a positive control of Gag processing, a protease inhibition event was detected in samples treated with nelfinavir at the concentration of 1 μM. However, samples treated with LPT12 did not show an accumulation of p25 as expected. It did not show the same inhibition pattern as nelfinavir since it did not increase the amount of p55. Instead, treatment with LPT12 showed a decrease in p24 processing in comparison to the non-treated-wild type virus positive control where both p25 and p24 are detected at higher concentrations (Figure 10). Results are shown as a representative one of three independent experiments.

### LPTs inhibits HIV-1 infection in PBLs

**Figure 11:**
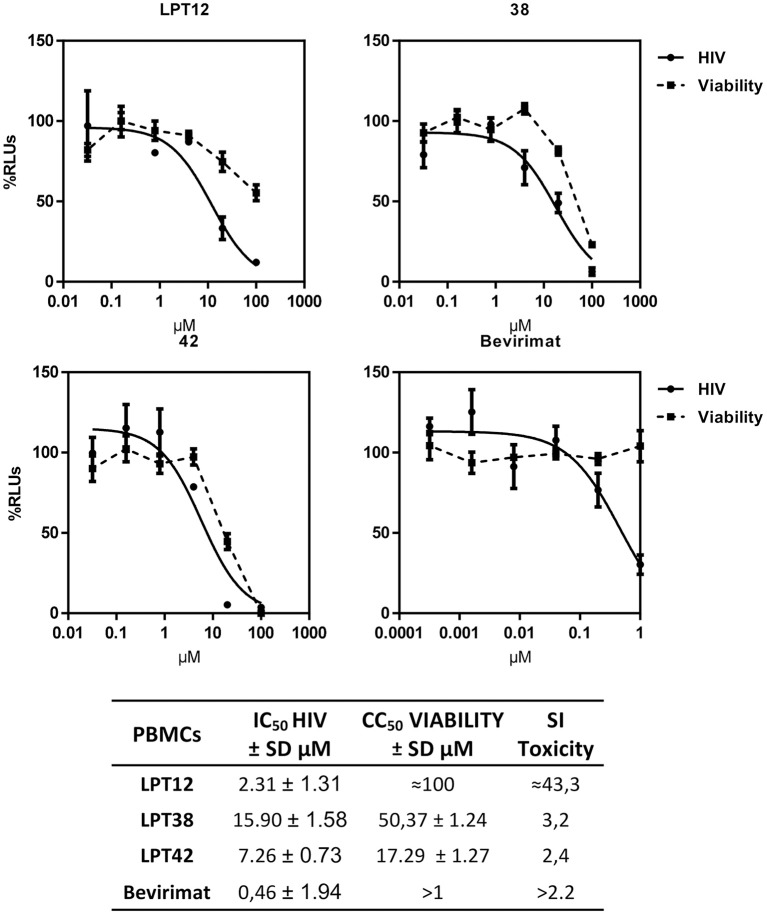
Dose response inhibition curves of HIV-1 (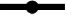
HIV) replication in primary human lymphocytes (PBLs) by LPT12, LPT38, LPT42, and bevirimat. Viability was measured in mock infected cultures (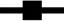
Viability). Results are shown as percentage of RLUs as compared to an untreated control. IC_50_ values were calculated with GraphPad Prism software, using non-linear regression analysis (Log inhibitor vs. response). SD, Standard deviation; SI toxicity, Specificity index toxicity (CC_50_/IC_50_).

Finally, we have tested the antiviral effect of LPTs in PBLs to corroborate their activity in the natural target of HIV-1, T cells. PBLs were isolated from healthy donors and pre-activated with IL-2 for at least 48 h before the experiment to allow HIV-1 infection. LPTs inhibited HIV-1 infection in PBLs, although some differences with MT-2 infections were found. While LPT12 and bevirimat displayed IC_50_ values similar to those shown in MT-2 infections, LPT38 and LPT42 increased their IC_50_ values approximately by a factor of 4. Toxicity values were higher in PBLs than in MT-2 for all the three LPTs, although LPT12 showed a CC_50_ higher than its IC_50_ (Figure 11). Results are the mean +/- SD of at least 3 independent experiments, each in triplicate.

## Discussion

Since the approval of raltegravir in 2007, antiretroviral therapy is mainly focused in the development of new formulations or combinations of currently approved drugs. However, HIV-1 infection is not cured by current therapy, but viral concentration is decreased to undetectable levels without eradicating the virus. Thus, new approaches are needed for the treatment of HIV-1 infections. Bevirimat was developed by chemical modification of betulinic acid, a lupane-type triterpene, as a new anti-HIV-1 candidate inhibitor of HIV-1 maturation (Sakalian et al., 2006). Maturation was, and still is, a drug target in HIV-1 lifecycle not reached by current ART and thus, maturation inhibitors have gained a considerable interest as potential new antiretrovirals. However, in clinical trials, bevirimat showed less activity than expected because of the presence of a natural mutation in Gag protein that conferred resistance to some strains of HIV-1 (Lu et al., 2011). Therefore, new derivatives of betulinic acid have been isolated or synthetized to overcome this resistance (Qian et al., 2009). In contrast to our investigations, these new derivatives have been tested only as maturation inhibitors. In this work, three powerful anti-HIV-1 inhibitors were selected from a set of previously screened lupane-triterpenoids (Callies et al., 2015) to study their potential as maturation inhibitors, as well as their activity on other targets in the HIV-1 replication cycle.

The chemical structure of the three LPTs is very similar, with slight variations in their substituents. Previous reports showed that betulinic acid derivatives with side chain modifications at the C_28_ position inhibited viral entry (Holz-Smith et al., 2001) targeting the V3 region of HIV-1 gp120 (Huang et al., 2006). Thus, betulinic acid displays other anti-HIV-1 activities besides maturation inhibition, and consequently, we have explored viral entry as a potential target of LPTs. LPTs inhibited infections with wild type HIV-1 and VSV-pseudotyped HIV-1 which enters T cells independently of CD4 and co-receptors, with similar IC_50_ values suggesting a non-entry target for all the LPTs and, of course, for bevirimat (Figure 2). Infection inhibition was also evident in primary human T cells (PBLs), ruling out a potential target in MT-2 cell line not related to regular T cells (Figure 11).

Furthermore, bevirimat was not able to inhibit HIV-1 infection completely, as shown in the graphs (Figure 2) for both MT-2 cells and PBLs at the concentrations tested. This can be explained because it targets a post-transcriptional step, viral maturation, and our recombinant virus (RV) system allows us to measure HIV-1 transcriptional activity (in RLUs). We have consistently found this post-transcriptional effect for protease inhibitors (Callies et al., 2015), as well as for saquinavir and lopinavir (Figure 8). Therefore, the first round of replication is detected if a post-transcriptional target is inhibited and RLUs would be obtained. However, the following rounds of replication will be inhibited and a decrease in RLUs would be detected, although RLUs from the first round will still be present. This peculiarity of our recombinant virus system leads us to recognize drugs with pre- and post-transcriptional targets by performing a simple infection experiment. Interestingly, LPTs inhibited HIV-1 replication completely and thus they likely have a pre-transcriptional target not related to maturation.

Several antiretrovirals act through inhibition of reverse transcription (RT), but little is known about the effect which triterpenes exhibit in this crucial step of the viral replication cycle. Rukachaisirikul et al. showed that protostane triterpenes inhibited RT activity in an enzymatic assay, although these compounds were cytotoxic in cell culture (Rukachaisirikul et al., 2003). Another study reported a potent anti-HIV-1 activity through anti- reverse transcriptase activity of secodammranane triterpenoids in a *times of addition* experiment. Moreover, this study showed that these triterpenes were able to inhibit other viruses such as simian immunodeficiency virus and murine leukemic virus (Esimone et al., 2010). Herein, we demonstrate that lupane-type triterpenoids could inhibit RT activity since LPT12 and LPT42 diminish the amount of viral DNA reverse transcripts produced in infected cells to the same extent than the reverse transcriptase inhibitor AZT. Interestingly, LPT38 treatment did not decrease total viral DNA amount so this might indicate the presence of an additional, unknown pre-transcriptional target. LPT12 did not inhibit reverse transcription at 10 μM, but it did at 20 μM, so it might also have another target in the HIV-1 replication cycle, since its infection IC_50_ is around 3 μM. On the other hand, LPT42 decreased viral DNA amount at a concentration of 2 μM, similar to its infection IC_50_ (1.6 μM).

Hence, two bevirimat-related compounds, LPT12 and LPT42, acted on a pre-transcriptional stage, inhibiting reverse transcription. This could explain the observation that these compounds were able to inhibit HIV-1 infection completely (Figure 2), while bevirimat did not with the concentrations tested. In contrast, LPT38 did not inhibit reverse transcription. There is no previous literature about the activity of lupane terpenes in HIV-1 integration. We found that two LPTs, LPT12, and LPT38, inhibited integration while LPT42 did not. The slight differences in their structure are crucial for the integrase inhibition. LPT38 decreased the number of integrated copies of HIV-1 as expected, since its antiviral activity is only observed at 20 μM and is thus likely to have another pre- or post-transcriptional viral target to reach its full activity.

LPTs are involved in HIV-1 transcription, as we have previously shown with oleanane triterpenes (Osorio et al., 2012). Again, slight structural differences of the LPTs lead to differences in their activity. In transcription, LPT42 is as powerful as in HIV-1 inhibition while LPT12 and LPT38 were not, suggesting that chemical features in LPT42 such as the hydroxyl or the acetate groups at the C-3 and C-28 positions, respectively, which could be able to act as H-bond acceptor/donor in the interaction with a receptor, are important trends for this activity (Figure 1). We described previously for oleanane terpenes that the overall oxidation level of the molecule, the regiosubstitution patterns, and the type of functional group, contributed to the inhibition of HIV-1 transcription (Osorio et al., 2012). This could be true also for LPTs, since LPT42 oxidation level in C-3 is different from LPT12 and LPT38, explaining in part its higher activity in HIV-1 transcription.

It has been previously described that coevolution of *gag* mutations with protease mutations could occur and patients who fails protease inhibitors (PIs) therapy could fail to respond to bevirimat therapy (Fun et al., 2011) probably because maturation involves the interaction with Gag HIV-1 protein in the protease cleavage site. Our findings show that all of them, including bevirimat, inhibited the amount of p24 Gag production. This fact is expected for two reasons: the inhibition of p24 production, with the subsequent accumulation of its precursor p25, is the described mode of action of maturation inhibitors and, secondly, transcriptional inhibition could diminish the amount of p24 production. The effect on protease activity was further studied by evaluation of LPTs activity in infections performed with HIV-1 resistant to PIs. We found that mutations that confer resistance to PIs did not modify the IC_50_ of LPTs and therefore, LPTs will be effective also in the infection with PIs resistant virus, at least with the mutations described for the viral strains used (M36I, I54V, L63P, A71V, G73S, and L90M).

We have used 3 different approaches to study LPTs activity as maturation inhibitors, the main target of bevirimat and some related lupane triterpenoids in HIV-1 cycle: evaluation of LPTs effect on viral particles production-maturation, LPTs activity in bevirimat resistant HIV-1 and LPTs activity on CA-SP1 processing (p25-p24). Maturation is a target not easy to study in a cellular environment, particularly if the compounds to analyze have more than one target in the replication cycle, since results can be interfered by their activity on previous targets. However, our results suggests low efficacy as maturation inhibitors for both LPT38 and LPT42. On the other hand, LPT12 clearly inhibited wild type HIV-1 maturation although, when a bevirimat resistant virus was used, LPT12 showed a slight susceptibility to the resistant virus, although no differences in IC_50_s were found in infections performed with wild type and bevirimat resistant HIV-1. This data indicate that the other targets in HIV-1 replication cycle, reverse transcription, integration and viral transcription, bypass the resistance to bevirimat. This fact was confirmed when CA-SP1 processing (p25-p24, Figure 10) was evaluated, since LPT38 and LPT42 did not show any effect in the p25/p24 ratio, confirming their low activity as maturation inhibitors and LPT12 activity was not clear in all the experiments performed. However, the three LPTs did not accumulate p55, which is a difference with the activity of protease inhibitors (nelfinavir) and, thus, LPTs target will not work in the same way as protease inhibitors.

In summary, three lupane triterpenoids structurally related to the well-known betulinic acid bevirimat were found to inhibit HIV-1 infection through more than one target. Thus, they act as promiscuous compounds by acting on multiple steps in the HIV-1 replication cycle in both MT-2 cells and PBLs. LPT12 inhibits HIV-1 infection, targeting reverse transcription, integration, viral transcription, Gag production and maturation. LPT38 also inhibits HIV-1 infection by targeting integration, viral transcription and Gag production. Finally, LPT42 targets reverse transcription, viral transcription and Gag production (Table 1). The multi-target profile of these compounds is a new feature of lupane terpenes that could be useful to overcome HIV-1 resistance by modulating multiple targets simultaneously. This approach, which plays an increasing role in pharmaceutical R&D, could facilitate the discovery of the next generation of anti-HIV-1 agents.

**Table 1:** Summary of the IC_50_ values (μM) and active concentrations obtained for LPT12, LPT38, LPT42, and Bevirimat in the different targets of the HIV-1 replication cycle studied.

**μM**		**LPT12**	**LPT38**	**LPT42**	**BVM**
HIV infection	MT-2	3.40 ± 1.76	4.08 ± 0.18	1.66 ± 0.17	0.009 ± 0.005
	PBLs	2.31 ± 1.62	15.90 ± 1.58	7.26 ± 0.73	0.008 ± 0.003
	PIs resistant HIV-1	2.70 ± 1.84	2.43 ± 2.86	7.07 ± 2.29	ND
	BVM resistant HIV-1	2.64 ± 0.03	5.30 ± 1.84	2.15 ± 0.37	4.18 ± 0.03
Reverse transcription^*^		20	>20	4	ND
Integration^*^		<10	≈20	>4	ND
HIV-1 transcription		11.6 ± 2.9	≫20	1.9 ± 0.9	>50
Gag-p24^*^		10	20	2	0.1
HIV-1 maturation	HIV-1^*^	10	20	2–8	≈0.1
	BVM resistant^*^	20	20	2–8	ND

* *Approximate values*.

## Datasets are available on request

The raw data supporting the conclusions of this manuscript will be made available by the authors, without undue reservation, to any qualified researcher.

## Author contributions

LB, IB, and JA: developed the original idea, designed the experiments and elaborated data; LB, MB, PO-C, IJ, OC, and JG-P: performed experiments and prepared figures; LB, JA, OC, IJ, and IB: edited and reviewed the final version of the article. All listed authors contributed to article writing.

### Conflict of interest statement

The authors declare that the research was conducted in the absence of any commercial or financial relationships that could be construed as a potential conflict of interest.

## Abbreviations

HIV-1: human immunodeficiency virus type 1
AIDS: acquired immunodeficiency syndrome
LPTs: Lupane-type pentacyclic triterpenoids
MI: maturation inhibitors
BA: betulinic acid
BVM: bevirimat
VSV: vesicular stomatitis virus
SD: standard deviation
SI: specificity index
CC_50_: cytotoxic concentration 50%
IC_50_: inhibitory concentration 50%
RT: reverse transcriptase
RAL: raltegravir
RLUs: relative luminescence units
PIs: protease inhibitors
NVF: nelfinavir
ART: antiretroviral therapy
PBMCs: peripheral blood mononuclear cells
PBLs: peripheral blood lymphocytes.
