# Supplementary material for: Promiscuous, Multi-Target Lupane-Type Triterpenoids Inhibits Wild Type and Drug Resistant HIV-1 Replication Through the Interference With Several Targets
Source: Front Pharmacol. 2018 Apr 18;9:358. doi: 10.3389/fphar.2018.00358 (PMC5915803; doi:10.3389/fphar.2018.00358)
Supplement: Supplementary file 1 [file Table1.docx]

Supplementary Material

PROMISCUOUS, MULTI-TARGET LUPANE-TYPE TRITERPENOIDS INHIBITS WILD TYPE AND DRUG RESISTANT HIV-1 REPLICATION TRHOUGH THE INTERFERENCE WITH SEVERAL TARGETS

**LM Bedoya*^1,3^, M Beltrán^1^, García-Perez J^1^, Obregón-Calderon P^1^, Callies O^2^, Jiménez IA^2^, Bazzochi IL^2^, J Alcamí*^1^**

^1^ AIDS Immunopathogenesis Department, Retrovirus Laboratory. National Centre of Microbiology, Instituto de Salud Carlos III, Madrid, Spain.

^2^ Instituto Universitario de Bio-Orgánica Antonio González, Departamento de Química Orgánica, Universidad de La Laguna, La Laguna, Tenerife, Spain.

^3^ Pharmacology Department, Pharmacy Faculty, Universidad Complutense de Madrid, Madrid, Spain.

*** Correspondence:**

José Alcamí PhD. MD.

[ppalcami@isciii.es](mailto:ppalcami@isciii.es)

Luis Miguel Bedoya PhD.

[lmbedoya@ucm.es](mailto:lmbedoya@ucm.es); [lmbedoya@isciii.es](mailto:lmbedoya@isciii.es)

# Supplementary Table S1. PCR thermal cycling conditions and primers and probes used in qPCR experiments to quantify reverse trancripts and integrated provirus

| **Reverse transcription** | | | |  | |  |  |  |
| --- | --- | --- | --- | --- | --- | --- | --- | --- |
| **HIV-1 Reverse transcription (early)** | | | MA pr-243 | | | GTGCCCGTCTGTTGTGTGAC | | |
|  |  |  | MA pr-244 | | | GGCGCCACTGCTAGAGATTT | | |
|  |  |  | MA pr-275 probe | | | CTAGAGATCCCTCAGACCCTTTTAGTCAGTGTGG (FAM) | | |
| **HIV-1 Reverse transcription (late)** | | | MH 531 | | | TGTGTGCCCGTCTGTTGTGT | | |
|  |  |  | MH 532 | | | GAGTCCTGCGTCGAGAGATC | | |
|  |  |  | Probe LRT-P | | | CAGTGGCGCCCGAACAGGGA (FAM) | | |
| **CCR5 gene** | | | CCR5_R | | | CTCACAGCCCTGTGCCTCTTCTTC | | |
|  |  |  | CCR5_F | | | GCTGTGTTTGCGTCTCTCCCAGGA | | |
|  |  |  | PROBE CCR5 | | | AGCAGCGGCAGGACCAGCCCCAAG (FAM) | | |
| **Integration** | | |  |  | |  |  |  |
| **1st PCR** | | | Primer Alu 1 | | | TCCCAGCTACTGGGGAGGCTGAGG | | |
|  |  |  | Primer Alu 2 | | | GCCTCCCAAAGTGCTGGGATTACAG | | |
|  |  |  | Primer L-M667 | | | ATGCCACGTAAGCGAAACTCTGGCTAACTAGGGAACCCACTG (FAM) | | |
| **2nd PCR** | | | Primer Lambda T | | | ATGCCACGTAAGCGAAACT | | |
|  |  |  | Primer AA55M | | | GCTAGAGATTTTCCACACTGACTAA | | |
|  |  |  | Probe MH603 | | | ACACTACTTGAAGCACTCAAGGCAAGCTTT (FAM) | | |
| **CCR5 gene** | | | CCR5_R | | | \| CTCACAGCCCTGTGCCTCTTCTTC \| \| --- \| | | |
|  |  |  | CCR5_F | | | GCTGTGTTTGCGTCTCTCCCAGGA | | |
|  |  |  | PROBE CCR5 | | | AGCAGCGGCAGGACCAGCCCCAAG (FAM) | | |
| All PCRs except Integration First PCR | | | | |  |  |  |  |
| 50 °C | 2:00 |  | | |  |  |  |  |
| 95 °C | 10:00 | Initial denaturation | | |  |  |  |  |
| 95 °C | 0:15 | 50 cycles | | |  |  |  |  |
| 60ºC | 1:00 |  |  |  |  |  |  |  |
| Integration First PCR | | | | |  |  |  |  |
| 95 °C | 8:00 | Initial denaturation | | |  |  |  |  |
| 95 °C | 1:00 | 12 cycles | | |  |  |  |  |
| 60 °C | 1:00 |  |  |  |  |  |  |  |
| 72 °C | 10:00 |  |  |  |  |  |  |  |
| 72 °C | 15:00 | Final extension | | |  |  |  |  |
